# Crossing the Boundary: No Catastrophic Limits on Infants’ Capacity to Represent Linguistic Sequences

**DOI:** 10.1111/desc.70015

**Published:** 2025-04-07

**Authors:** Natalia Reoyo‐Serrano, Anastasia Dimakou, Chiara Nascimben, Tamara Bastianello, Daniela Lucangeli, Silvia Benavides‐Varela

**Affiliations:** ^1^ Department of Developmental Psychology and Socialisation University of Padova Padova Italy; ^2^ Padova Neuroscience Center University of Padova Padova Italy

**Keywords:** auditory discrimination, boundary effect, language processing, number representation, syllables

## Abstract

The boundary effect, namely the infants’ failures to compare small and large numerosities, is well documented in studies using visual stimuli. The prevailing explanation is that the numerical system used to process sets up to 3 is incompatible with the system employed for numbers >3. This study investigates the boundary effect in 10‐month‐old infants presented with linguistic sequences. In Condition 1 (2 vs. 3), infants can differentiate small syllable sequences (2 vs. 3), with better performance for the 2‐syllable sequence, which imposes a lower memory load. Condition 2 (2 vs. 4) revealed that infants are capable of discriminating across bounds, with relatively higher performance for the 4‐syllable sequence, possibly encoded as one large ensemble. This study offers evidence that, when processing linguistic sounds, infants flexibly deal with small and large numerical representations with no boundaries or incompatibilities between them. Simultaneously encoding units of different magnitudes might aid early speech processing beyond memory limits.

## 1 Introduction

Under natural circumstances, our auditory perception is rarely chaotic or misleading. Even infants in their first months of life have the capacity to parse the acoustic input into auditory perceptual units (Benavides‐Varela et al. 2012; Hochmann and Papeo 2014; Räsänen et al. 2018), extract featural information of the temporarily distributed elements (Benavides‐Varela and Gervain 2017; Benavides‐Varela and Mehler 2015; Gómez et al. 2014; Fló et al. 2019), generate predictions for upcoming sounds in the auditory scene (Fló 2021; Fló et al. 2022), and even for elements in other modalities (Brower and Wilcox 2012; Robinson and Sloutsky 2008). Such remarkable abilities contribute to a coherent perception of the auditory input and lean on the core capacity to store multiple sounds and compute relations among them simultaneously. The present study sought to explore this early capacity, focusing on the infants’ ability to represent and extract a fundamental feature, namely the number of syllables from auditory streams. Specifically, we investigated the properties of the numerical representations supporting small and large numbers of linguistic sounds.

**Summary**

- Ten‐month‐old infants successfully discriminate 2‐ versus 3‐syllable and 2‐ versus 4‐syllable sequences in a working memory (WM) task.
- This suggests no boundary effect with linguistic stimuli, unlike with visual stimuli.
- Infants find it easier to process 2‐syllable sequences compared to 3‐syllable sequences, while the opposite is true when comparing 2‐syllable sequences to 4‐syllable sequences.
- Infants possess compatible and flexible mechanisms enabling them to maintain units of different magnitudes, even those exceeding their WM capacities.

Influential views posit the existence of innate, evolutionary ancient cognitive systems that support early numerical cognition in human and nonhuman species (Butterworth 2005; de Hevia 2016; Dehaene 2011; Dehaene et al. 1998; Di Giorgio et al. 2019; Kobylkov et al. 2023; Nieder and Dehaene 2009; Rugani et al. 2024; Rugani et al. 2020) (for recent reviews see Butterworth 2022; Visibelli et al. 2024). Studies showed that preverbal infants are sensitive to numerosity differences in both small (e.g., 1 vs. 3) and comparatively large sets (e.g., 8 vs. 16) when employing visual and audio–visual tasks (Coubart et al. 2014; Feigenson, Carey, and Spelke 2002; Izard et al. 2009; Libertus et al. 2014; Libertus and Brannon 2010; Martin et al. 2022). This early sensitivity is thought to be supported by two separate cognitive systems as follows: (a) a parallel individuation or object tracking system (OTS), which represents individual objects in small sets (generally three or fewer) in a precise way, and (b) the Approximate Number System (ANS), which encodes larger numerosities in an approximate, ratio‐dependent way (Feigenson and Carey 2005; Feigenson, Carey, and Hauser 2002; Feigenson, Carey, and Spelke 2002; Hyde 2011; Lipton and Spelke 2004; Xu 2003; Xu et al. 2005).

### 1.1 Discrimination and catastrophic failures in the visual modality

The functioning of the small number system is apparent since birth (Martin et al. 2022); however, its capacity is severely limited (Coubart et al. 2014). Infants, like adults, effortlessly perceive the exact number of elements when fewer than three (or two) objects are displayed, which is about the limit in working memory capacity (Cowan 2016, 2022; Endress and Szabó 2017; Knops et al. 2014). Once this threshold is surpassed, adults use counting to determine the number of items with precision or simply resort to approximate estimations (Barth et al. 2003; Benavides‐Varela et al. 2018; Izard and Dehaene 2008). Conversely in infants, once this boundary is exceeded, catastrophic failures are generally observed such that infants not only fail to recognize the presence of more than 1 object in the set, but they even fail to remember any of the previously presented items (Coubart et al. 2014, 2015; Feigenson and Carey 2003, 2005; Feigenson, Carey, and Hauser 2002; Feigenson, Carey, and Spelke 2002). For instance, when presented with crackers being distributed into two separate containers, infants aged 10–12 months consistently crawl towards the container with the greater number of crackers when one container contains 1 cracker and the other holds 2 or 3 crackers. However, when one container holds a small quantity of crackers while the other contains a larger amount (2 vs. 4, 1 vs. 4, 3 vs. 6), infants make random choices between the containers (Feigenson and Carey 2003, 2005; Feigenson, Carey, & Hauser 2002). Moreover, in a modified visual change‐detection paradigm, 10‐month‐old infants detect feature changes in arrays containing 2 or 3 individual objects but fail with arrays containing 6 (Ross‐Sheehy et al. 2003). In a manual search paradigm, 12–14‐month‐old infants successfully remember the hiding of 1, 2, or 3 individual objects in an opaque box but fail when 4 objects are hidden (Barner et al. 2007; Feigenson and Carey 2003, 2005). The most accepted explanation of these catastrophic failures is that the number system used to process small numbers up to three (OTS) is incompatible with the system used to process large numbers beyond three (ANS) (Feigenson, Carey, and Hauser 2002; Feigenson and Carey 2005; Hyde 2011; Mou and Van Marle 2014; Posid and Cordes 2015). Since the two number systems exhibit mutual incompatibility, infants lack the means to compare the numerosities of elements below 3 with those exceeding 3.

### 1.2 Discrimination in the auditory modality

The capacity to represent the number of multiple sounds has been considerably less studied in the auditory modality. The existing studies yield a more complex scene regarding the operation of the two numerical representational systems.

In a pioneering study, Bijeljac‐Babic et al. (1993) examined whether 4‐day‐old infants could detect changes in the number of syllables in multisyllabic utterances. They controlled for the total sound duration and used the high‐amplitude sucking procedure. The results revealed that infants, following habituation, increased their sucking rate when exposed to a different number of syllables (i.e., 2 vs. 3 CV‐syllables). The study demonstrated that newborns distinguish linguistic elements organized at the syllabic level in the small number range. Their work also provided evidence that, when it comes to large numerosities, ANS acuity is limited and does not yet permit a 2:3 ratio discrimination, since newborns did not respond to changes in the number of individual phonetic segments (i.e., 4 vs. 6 phonemes). As demonstrated by other studies in the visual domain, neonates require a broader ratio (1:3) to successfully discriminate between sets (Coubart et al. 2014; Izard et al. 2009).

In another study, Lipton and Spelke (2004) provided evidence that 6‐month‐old infants discriminate among large numbers of sounds (bells, whistles, chirps, buzzes, drums, and horns) differing by a 1:2 ratio (i.e., 8 vs. 16 and 4 vs. 8 sounds). Moreover, in agreement with other small‐large discrimination failures of visual sets, infants could not discriminate between small and large numbers of sounds (e.g., 2 vs. 4). This could have been taken as evidence that incompatibilities between the ANS and OTS do exist in the auditory, as in the visual domain. However, the study also showed that 9‐month‐old infants were not able to discriminate between small numbers of sounds (i.e., 2 vs. 3). Thus, while the success in discriminating large sets of sounds was interpreted as proof of the infants’ capacity to access the ANS, the failure to discriminate 2 versus 3 or 2 versus 4 suggested, according to the authors, that neither the OTS nor the ANS were engaged when processing nonlinguistic sounds in the small number range. The authors proposed that the OTS might not be available to infants when processing auditory elements because it is functional for representing visual elements only. In a following study, van Marle and Wynn (2009) demonstrated that 7‐month‐olds successfully detect the difference between small and large numbers of tones (2 and 4) but not between small numbers of tones (2 and 3). These results do not resemble the incompatibilities between ANS and OTS found with visual sets. Accordingly, they were taken as evidence that, although infants might not have access to OTS, they can use approximate and ratio‐dependent representations of the ANS to discriminate across small and large numbers of sounds, in this case, tones. Subsequently, Benavides‐Varela and Reoyo‐Serrano (2021) provided evidence that, under certain circumstances, auditory representations could be more precise. In this study, 9–10‐month‐old infants were capable of precisely representing and distinguishing small sets (2 and 3), but only when exposed to sounds of a linguistic nature (consistent with the findings of Bijeljac‐Babic et al. 1993). Using the same paradigm, infants were not able to distinguish 2 versus 3 sequences of tones, like in the studies of Lipton and Spelke (2004) and van Marle and Wynn (2009).

Altogether, the above‐cited evidence, even if limited, suggests that different representational mechanisms operate when listening to sounds of a varied nature. While tones and other nonlinguistic sounds facilitate approximate ratio‐dependent representations which gradually increase in precision with age, linguistic sounds also provide access to a precise representational system within the small number range (see Table 1)^1^. This precision is not surprising, considering that language is especially effective in infancy for triggering early mechanisms of attention (Marno et al. 2015; Saksida and Langus 2024; Vouloumanos and Curtin 2014; Vouloumanos and Werker 2004, 2007), categorization (Ferry et al. 2010), abstract relations (Hochmann 2022), memory (Benavides‐Varela et al. 2017; Benavides‐Varela, Gómez, Macagno et al. 2011, Benavides‐Varela, Gómez, and Mehler 2011), and for highlighting properties that might be otherwise difficult to detect or encode.

**TABLE 1:** Summary of the current literature on numerical discrimination based on sounds, tones, and syllables. It shows that nonlinguistic sounds provide access to approximate ratio‐dependent representations, which gradually increase in precision with age, while linguistic sounds enable precise representations, particularly within the smaller number range.

Study	Age	Method	Type of stimuli	Controlled features	Small quantities	Intermediate quantities	Large quantities
Bijeljac‐Babic et al. (1993)	4 Days old	High‐amplitude sucking procedure	*Linguistic* CV‐syllable sequences	—	2 vs. 3 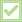	—	—
	4 Days old	High‐amplitude sucking procedure	*Linguistic* CV‐syllable sequences	– Stimuli duration	2 vs. 3 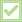	—	—
	4 Days old	High‐amplitude sucking procedure	*Linguistic* Phoneme sequences	– Number of syllables	—	—	4 vs. 6 (2:3) 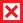
Lipton and Spelke (2004)	6 Months old	Head‐turn preference procedure	*Nonlinguistic* Sound sequences	– Single sound duration – Sequence duration – Sequence rate – Amount of total acoustic energy – Interstimulus intervals	—	2 vs. 4 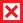	4 vs. 8 (1:2) 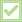
							4 vs. 6 (2:3) 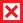
	9 Months old	Head‐turn preference procedure	*Nonlinguistic* Sound sequences	– Single sound duration – Sequence duration – Sequence rate – Amount of total acoustic energy – Interstimulus intervals	2 vs. 3 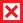	—	4 vs. 6 8 vs. 12 (2:3) 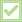
							4 vs. 5 (4:5) 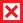
van Marle and Wynn (2009)	7 Months old	Habituation	*Nonlinguistic* Tone sequences	– Melodic properties – Continuous temporal properties	2 vs. 3 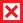	2 vs.4 (2:4) 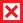	—
Benavides‐Varela and Reoyo‐Serrano (2021)	9–10 Months old	Preferential looking paradigm	*Linguistic* CV‐syllable sequences	– Duration of each syllable – Pitch – Intensity – Interstimulus duration – Rate of syllable presentation	2 vs. 3 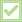	—	3 vs. 4 (3:4) 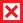
	9–10 Months old	Preferential looking paradigm	*Linguistic* CV‐syllable sequences	– Duration of the sequences – Pitch – Intensity – Interstimulus duration – Rate of syllable presentation	2 vs. 3 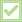	—	—
	9–10 Months old	Preferential looking paradigm	*Nonlinguistic* Tone sequences	– Duration of each tone – Pitch – Intensity – Interstimulus duration – Rate of syllable presentation	2 vs. 3 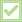	—	—

### 1.3 The present study

Despite this remarkable ability that brings, in terms of accuracy, linguistic representations in the small number range closer to representations of visual objects, other properties of the numerical representation elicited by speech units remain unclear. An intriguing yet still open question concerns whether the catastrophic nature and incompatibilities between the number systems used to process small and large numbers can also be observed in the auditory domain.

We investigated this question in 10‐month‐old monolingual infants, building on a two‐alternative‐looking paradigm previously used to test various cognitive abilities in infants (Addyman and Mareschal 2010; Albareda‐Castellot et al. 2011; Benavides‐Varela and Mehler 2015; Hochmann et al. 2011; Hochmann et al. 2018; Kovács and Mehler 2009; Kovacs and Mehler 2009; McMurray and Aslin 2004), including auditory number discrimination (Benavides‐Varela and Reoyo‐Serrano 2021). This paradigm involves familiarization and test trials. During familiarization trials, infants are presented with two kinds of stimuli intermixed (e.g., sequences of 2‐ and 3‐syllable sets) that predict the subsequent appearance of a puppet on either the left or right side of the screen (e.g., puppet appearance on the left side following the 2‐syllable sets and on the right side following the 3‐syllable sets). In the test, no puppet appears after the speech sequence. Infants might reliably look to the correct side of the screen only if they could (a) grasp the feature (i.e., number of sounds) that differentiates the multisyllabic sequences and (b) remember that this feature determined the toy's location in the previous phase.

In the two alternative‐looking paradigms, monolingual‐learning infants—like those tested in the present work—have trouble learning two regularities simultaneously (Hochmann et al. 2011; Kovacs and Mehler 2009). Thus, successful discrimination is indexed when infants’ performance is above chance in one of the two simultaneously presented sets, the one they find easier to process.

In Condition 1, we assessed infants’ ability to discriminate between syllable sequences within the small number range (2 vs. 3). In Condition 2, we tested them with one set within the small number range and another set that lies outside the small‐range bounds (2 vs. 4). Based on previous literature (Benavides‐Varela and Reoyo‐Searrano 2021), we expected infants to succeed in Condition 1. Moreover, we expected them to fail in Condition 2 if, as in the visual domain, the precise representations originating from the system used to process a small number of syllables are incompatible with the one used to represent a large number of syllables.

## 2 Materials and Methods

One experiment with two conditions was performed using a within‐subjects design in two separate days. In Condition 1, both syllable sequences were within the small number range (2 vs. 3), whereas in Condition 2, one set of sequences lies within the small number range (2), and the other one lies outside the small range bounds (4). The order of exposure to the conditions was randomized so that half of the participants were exposed to the 2‐ versus 3‐syllable sequences the first day, while the other half to the 2‐ versus 4‐syllable sequences.

### 2.1 Participants

A group of 25 monolingual Italian learning infants participated in the study (13 females; *M*_age_ = 10 months and 12 days, range 9 months 6 days to 11 months). The inclusion criteria considered only full‐term infants with no birth complications, no sensory or neurological problems, or familial language disorders. Seven additional infants were excluded from the analysis because of equipment failure (*N* = 1), their eye movements were not visible at the time of coding (*N* = 1), and they looked to one side before hearing the entire sequence of syllables during the test (*N* = 3), or they did not provide data on sufficient test trials (i.e., less than 50% of the trials; *N* = 1). Moreover, one participant did not provide enough data (i.e., contributed in less than 50% of the trials) during the familiarization phase of the first experiment. Following previous studies with the two‐alternative‐looking paradigm (e.g., Kovács and Mehler 2009; Kovacs and Mehler 2009), fixations lasting less than ≤80 ms were excluded from the analysis. See also the Supplementary Materials for considerations on side biases.

The final sample size was based on a power analysis considering the lowest effect size reported in the study of Benavides‐Varela and Reoyo‐Serrano (2021) testing 2‐ versus 3‐syllable sequences—Experiment 2 effect size = 0.52. The calculation was settled to achieve power = 0.80, and alpha = 0.05 in a one‐sample *t*‐test. The computation (G*power 3.1.9.6, Faul et al. 2007) indicated that a sample size of 25 participants was required.

Participants were recruited and tested online. Letters, online pamphlets, and social media advertisements were sent to parents from different areas of Italy (North, South, and Center). All participants who adhered to the study were Caucasians from rural and urban areas of Italy. The socio‐economic status of the participating families was middle‐high (21 out of 25), and it was measured through the educational level of the parents (19 out of 25 hold a university degree) and their working status. After accurately reading the whole procedure and all the issues presented in the informed consent, parents signed it digitally. The consent form was provided before the experiment started and included parents’ willingness to participate, provide demographic information, and access to audio–video recordings. The research protocol was approved by the members of the Ethical Committee of Psychology Research at the University of Padua (Protocol N. 3697).

### 2.2 Stimuli

The stimuli consisted of audio (i.e., linguistic) and visual items. Linguistic stimuli were either two‐, three‐, or four‐CV syllable sequences. CV syllables were constructed by pairing one consonant and one vowel. MBROLA Italian database IT4 (Dutoit et al. 1996) was used to synthesize the syllables with the female voice using eSpeak NG (https://github.com/espeak‐ng/espeak‐ng/). The syllables were constructed by combining six consonants (3 stop: /k/, /p/, /d/; 2 fricatives: /f/, /s/; and 1 lateral: /l/) selected based on their frequency in the first words in Italian and early age of acquisition (Keren‐Portoy et al. 2009; Romani et al. 2017), and two vowels (/a/, /u/) chosen because they are readily distinguished by young infants (Kuhl et al. 1997). The resulting ten syllables [ka, pa, sa, la, fa, ku, pu, su, du, fu] served to generate sixty different syllable sequences (see Supplementary Materials and Open Practices to access the stimuli). Sequences containing C‐/a/ syllables were presented to half of the infants, and sequences containing C‐/u/ were presented to the other half. The same set of syllables was used for the 2‐, 3‐, and 4‐syllable sequences to prevent infants from creating categories. For each participant, twelve different syllable sequences were used in the familiarization trials, and eight new syllable sequences were used in the test trials. All syllables created for the familiarization and test phases were randomized across participants.

The intensity (70 dB) and pitch (240 Hz) were kept constant in all the sequences. The token and sequence duration varied across trials so that infants could not use rhythm or duration differences to distinguish between the two‐syllable sequences. In half of the trials, the rhythm was the same in the two‐syllable sequences, with each syllable being 200 ms and the interstimulus interval (ISI) lasting 250 ms. In the remaining trials, the sequences were edited to have the same overall duration. Table 2 shows an overview of the features of the stimuli used in the two conditions. The syllable sequences were created using Audacity Cross‐Platform Sound Editor, version 3.1.3.

**TABLE 2:** Properties of the stimuli used in the two experiments.

Syllables sequences—Conditions						
Condition	Stimuli properties					Graphical example
	Sequence elements		Token duration	ISI duration	Sequence duration	
1	2 Syllables	CV–CV	200 ms	250 ms	650 ms	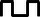
	3 Syllables	CV–CV–CV	200 ms	250 ms	1100 ms	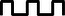
	2 Syllables	CV–CV	269 ms	337 ms	875 ms	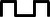
	3 Syllables	CV–CV–CV	159 ms	199 ms	875 ms	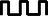
2	2 Syllables	CV–CV	200 ms	250 ms	650 ms	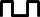
	4 Syllables	CV–CV–CV–CV	200 ms	250 ms	1550 ms	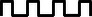
	2 Syllables	CV–CV	338 ms	423 ms	1100 ms	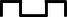
	4 Syllables	CV–CV–CV–CV	142 ms	177 ms	1100 ms	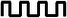

The visual stimuli consisted of one central attractor and six different cartoons presented one at a time inside one of the two white squares (one on the left and one on the right). The white squares measured 8 cm and on a 13″ computer, they appeared as 13.5 cm distant from one another. The 2‐s cartoons consisted of a puppet looming on the screen (from 4 to 7 cm). A 300‐ms tinkling bell appeared 800 ms after the onset of the visual stimulus. The colorful cartoons were used as visual reinforcement and randomly paired with the syllable sequence in the familiarization phase.

### 2.3 Procedure

**FIGURE 1:**
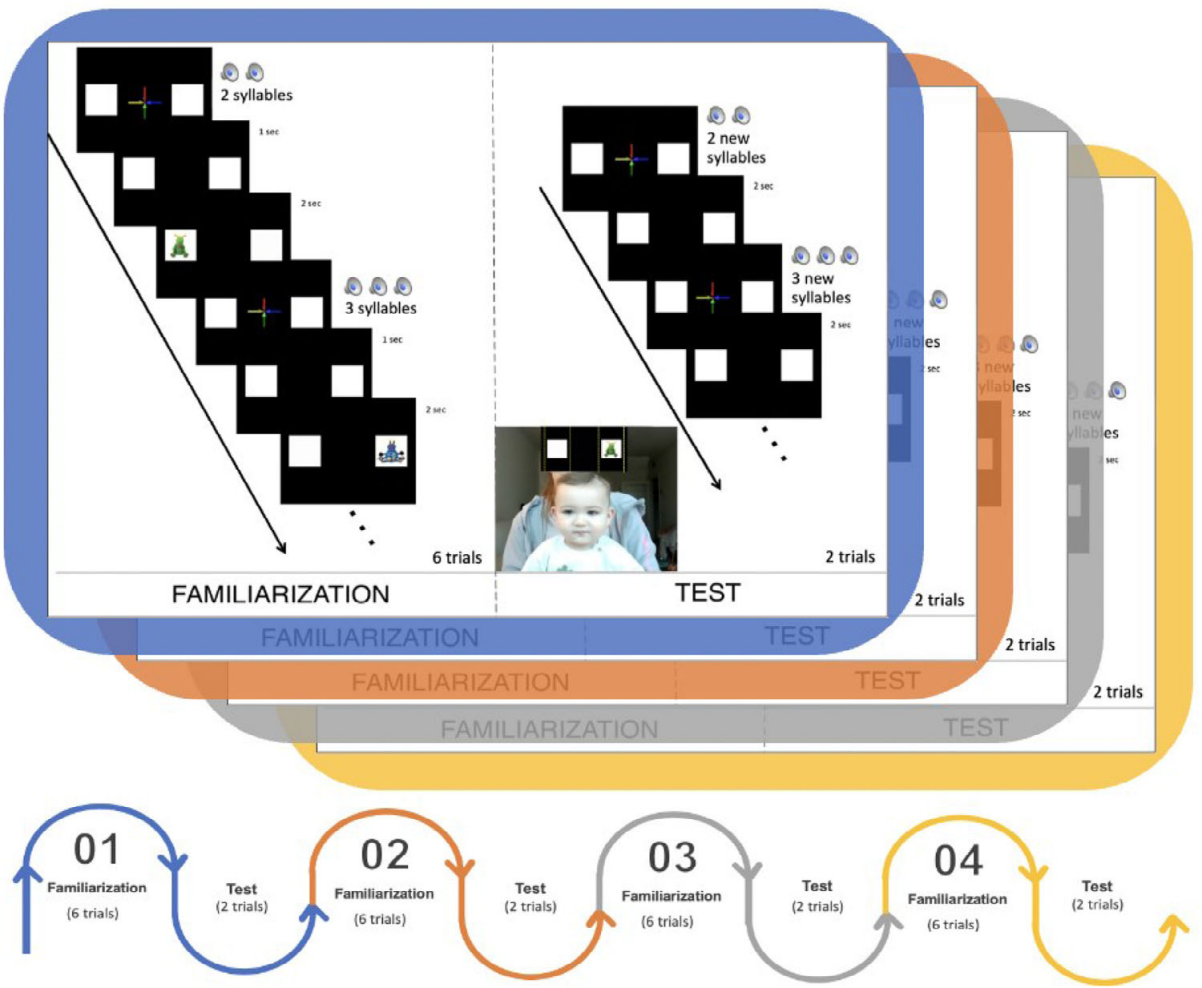
Illustration of the experimental procedure and timeline. At the bottom, the flow of the entire experiment is shown, highlighting the structure of each block (each color represents one block). In the upper part, inside the blue square, the flow for each familiarization and test trial is depicted. The screenshot showing the child illustrates the online setup.

The experiment consisted of a two‐alternative‐looking paradigm with blocks of 6 familiarization and 2 test trials each. The blocks were repeated four times, yielding 24 familiarization trials and 8 test trials (Figure 1). This paradigm has been implemented in previous studies to test various cognitive abilities in infants (Addyman and Mareschal 2010; Albareda‐Castellot et al. 2011; Benavides‐Varela and Reoyo‐Serrano 2021; Hochmann et al. 2011; Hochmann et al. 2018; Kovacs and Mehler 2009; Kovács and Mehler 2009; McMurray and Aslin 2004). The current adaptation aims to reduce dropouts and possible extinction effects caused by the successive presentation of test trials without reinforcement.

Familiarization trials were characterized by a visual attractor in the center of the screen placed between two white squares on the left and right sides. After 1.5 s, a syllable set was played. The attractor disappeared at the end of the auditory sequence, leaving only the two white squares visible. In one of these, a looming cartoon appeared for 2 s. In each condition, there were two‐syllable sequences. Condition 1 tested infants’ ability to distinguish 2‐ versus 3‐syllable sequences. Thus, a 2‐syllable sequence preceded the puppet's appearance in one of the squares, whereas a 3‐syllable sequence anticipated the puppet's appearance in the opposite square.

These sequences were presented in an interleaved pseudorandom order, ensuring that each was presented no more than twice in a row. Moreover, the presentation of specific sequences in the first trial was counterbalanced across blocks. Additionally, each of the two‐syllable and three‐syllable sequences was randomly presented, so infants always heard different stimuli and saw different pseudowords‐puppet associations. No cartoon was displayed in the test trials (one for each syllable sequence in each block). Thus, the only difference compared to the familiarization trials was that only the two white squares were shown for 2 s after the syllable sequences were displayed. After these 2 s, a new test trial started.

The order of the two conditions was counterbalanced across participants. Specifically, thirteen infants saw the 2 versus 3 condition first, while twelve saw the 2 versus 4 condition first. The two conditions were conducted in separate sessions, with an average interval of 5.48 days between them (range: 2–8 days). The side of appearance of the puppet was instead fixed: It always appeared on the left following the two‐syllable sequence, and on the right for the 3‐ or 4‐syllable sequences. Having infants associating the small sequence with the left and the large sequence with the right side is congruent with mental number line representations in Western cultures, which have been reported in infants and even newborns (Di Giorgio et al. 2019; Eccher et al. 2025). Inverting the side of the association could have potentially interfered with the aims of the study.

### 2.4 Data Acquisition

The data were collected online using Labvanced (https://www.labvanced.com/). To ensure methodological rigor and in line with current recommendations for online data collection (Bánki et al. 2022; Kaduk et al. 2024; Zaadnoordijk and Cusack 2022), an instruction session was offered to parents via Zoom. The session was carried out in the absence of the infant a few minutes before the test. To ensure adequate data quality, various measures were considered: the parents were asked to use a personal computer (no tablets or cell phones) with a screen size larger than 13″ and to choose a luminous and quiet environment in the house to run the experiment. The calibration procedure embedded in Labvanced was used to ensure that the visual stimuli appeared identical across computers of different sizes. The infant's distance from the screen was 50 cm. An app (speedtest.net) was used to test the stability of the internet connection. Instead, the quality and intensity of the sound were checked using parents’ cell phones, through Niosh for Apple and Fonometro for Android. Lastly, parents were required to switch off any additional program or app on their computer, turn on the silent mode on their cell phones, turn off any other electronic machines in the house (e.g., TV, radio), and remove toys or other objects from the infant's sight which could interfere with the test and attract their attention.

During the test sessions, the experimenter and the parent joined a Zoom session (see Open Practices for the extended protocol). At the beginning of this session, parents were requested to provide verbal consent (in addition to the written consent sent by email before the test). Subsequently, they were guided for audio and video adjustments and baby setup. Parents were invited to open the Labvanced link on Google Chrome, share their screen, and allow the experimenter to have remote control of their laptop. This procedure not only ensured that the experiment was presented in full‐screen mode to the child without any interference from Zoom video and control panels but also allowed the experimenter to control the execution of the experiment in Labvanced for the whole duration of the test. The session was recorded by the experimenter, and the progress of the testing was controlled through the use of specific computer keys to attract the infant's attention to the center of the screen or to start playing the syllables. Additionally, infants’ gaze was video‐recorded automatically via Labvanced.

### 2.5 Reliability

Two independent coders performed an offline analysis of all the videos by coding infants’ eye movements frame‐by‐frame. The correlation between observers was computed across dependent variables on 100% of the sample. Pearson's *r* = 0.92, *p* < 0.001, suggesting no significant differences between the blind coders. Video coding was carried out using the VirtualDub 1.10.4 program.

### 2.6 Data Analysis

The direction of the first look in each test trial was the primary measure, and it was determined by splitting the screen into two portions of equal size: left and right (see Figure 1). Infants’ fixations to the screen's left or right side were coded as correct or incorrect based on the auditory sequence previously presented. For example, if a sequence containing 2 syllables predicted the appearance of the cartoon on the right square during the familiarization, searching for the puppet on the right side of the screen after hearing the 2‐syllable sequence in the test was coded as correct. Otherwise, the trial was coded as incorrect.

The difference score was considered as the main dependent variable. This score was retrieved from the accuracy of the first‐looking behavior within the 8 test trials. It results from subtracting the number of trials in which the infant looked to the correct side of the screen from the number of incorrect trials. The result of such a difference was then divided by the number of total valid trials (either correct or incorrect).

$$\begin{array}{ccc} & & \text{Normalized difference score}\;\\ & & \;=\frac{\left(\#\text{correct trials}\;-\#\text{incorrect trials}\right)}{\left(\#\text{total trials}\right)} \end{array}$$

The score obtained from this formula could result in a value between −1 and 1, where the positive sign indicates correctness and the negative sign incorrectness. When the scores assumed a value around zero, these suggest that infants show no consistent pattern of responses and were at chance on average. Trials in which the first look started before the auditory sequence ended were excluded from the analyses.

For each of the two sequences, a series of one‐sample *t*‐tests were run to compare infants’ performance against chance. One‐tailed tests were computed reflecting the directional hypotheses that infants would perform above chance if successful discrimination was attained. A paired sample *t*‐test was conducted to test differences in the infant's performances in the two sequences.

The mean durations of looking times throughout the experiment were calculated and reported for each study. In addition, the latency of the responses and the proportion of trials on which infants fixated the screen were used to control for data quality.

Two additional indices of infants’ looking behavior were analyzed in the test phase. First, the proportion of time the participant looked to the correct side out of the total time looking at the screen in each trial (i.e., the proportion of correct looking time). Second, the longest look corresponds to the look towards one side of the screen with the longest duration. Then, similarly to the first‐look behavior, difference scores and relative statistical analyses were computed both for the proportion of correct looking and longest look indices. The looking behavior of the participants was separately coded for the familiarization phase. The results are presented in the Supplementary Materials.

## 3 Results

### 3.1 Condition 1: Discrimination of 2‐ and 3‐Syllable Sequences

**FIGURE 2:**
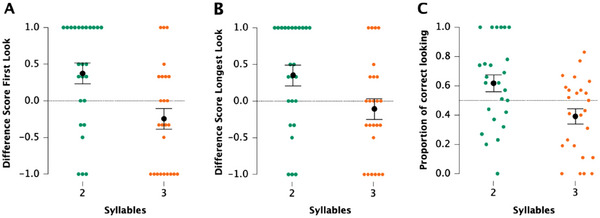
Main results of Condition 1—infant's ability to discriminate between sets of 2 and 3 syllables. These findings are summarized as follows: (A) Normalized difference scores based on first‐look duration. (B) Individual distribution of the normalized difference scores calculated on the longest look: Colored dots represent individual participants; black dots indicate the group means, and bars the standard error. Chance is determined by the dotted black line in the middle. (C) Proportion of gaze duration (correct side).

The results obtained in the test trials of this condition are depicted in Figure 2.

Analyses were carried out over three different dependent variables to assess the congruency of the infants’ behavior. The analysis considering the first look showed that, in the two‐syllable sequences, the infants’ mean difference score was 0.37. This was significantly greater than chance [*t*(24) = 2.63; *p* = 0.01; Cohen's *d* = 0.53]. For the three‐syllable sequences, the infants’ mean difference score was −0.24 and did not differ significantly from chance [*t*(24) = −1.70; *p* = 0.1; Cohen's *d* = −0.34]. Infants obtained higher scores in the 2‐syllable compared to those in the 3‐syllable sequences [*t*(24) = 2.39; *p* = 0.013; Cohen's *d* = 0.48]. Infants fixated on either side of the screen 80% of the time. The first looking duration was 660 ± 229 ms on average, the longest look was 765 ± 238 ms (on average), and the mean response latencies were 787 ± 236 and 777 ± 316 ms for the 2‐ and 3‐syllable sequences, respectively.

Results are similar when considering the longest‐look variable. In the two‐syllable sequences, infants’ mean difference score (0.35) was significantly greater than chance [*t*(24) = 2.47; *p* = 0.02; Cohen's *d* = 0.50], whereas in the three‐syllable sequences, infants’ mean difference score (−0.11) did not differ significantly from chance [*t*(24) = −0.78; *p* = 0.44; Cohen's *d* = −0.15]. Infants’ ability to detect the correct side of the screen is greater when presented with two‐syllable sequences compared to the three‐syllable sequences [*t*(24) = 1.85; *p* = 0.03; Cohen's *d* = 0.37].

The analysis considering the proportion of correct looking revealed findings similar to those of the previous analysis. The proportion of correct looking was significant for the 2‐syllable sequences [mean = 0.62; *t*(24) = 2.17; *p* = 0.04; Cohen's *d* = 0.43], and it was marginally significant for the 3‐syllable trials [mean = 0.39; *t*(24) = −2.09; *p* = 0.05; Cohen's *d* = −0.42]. A paired sample *t*‐test confirmed that infants looked differently to the correct side for the 2‐ versus 3‐syllable sequences [*t*(24) = 2.37; *p* = 0.01; Cohen's *d* = 0.47].

The results obtained in the familiarization trials are presented in the Supplementary Materials.

### 3.2 Condition 2: Discrimination of 2‐ and 4‐Syllable Sequences

**FIGURE 3:**
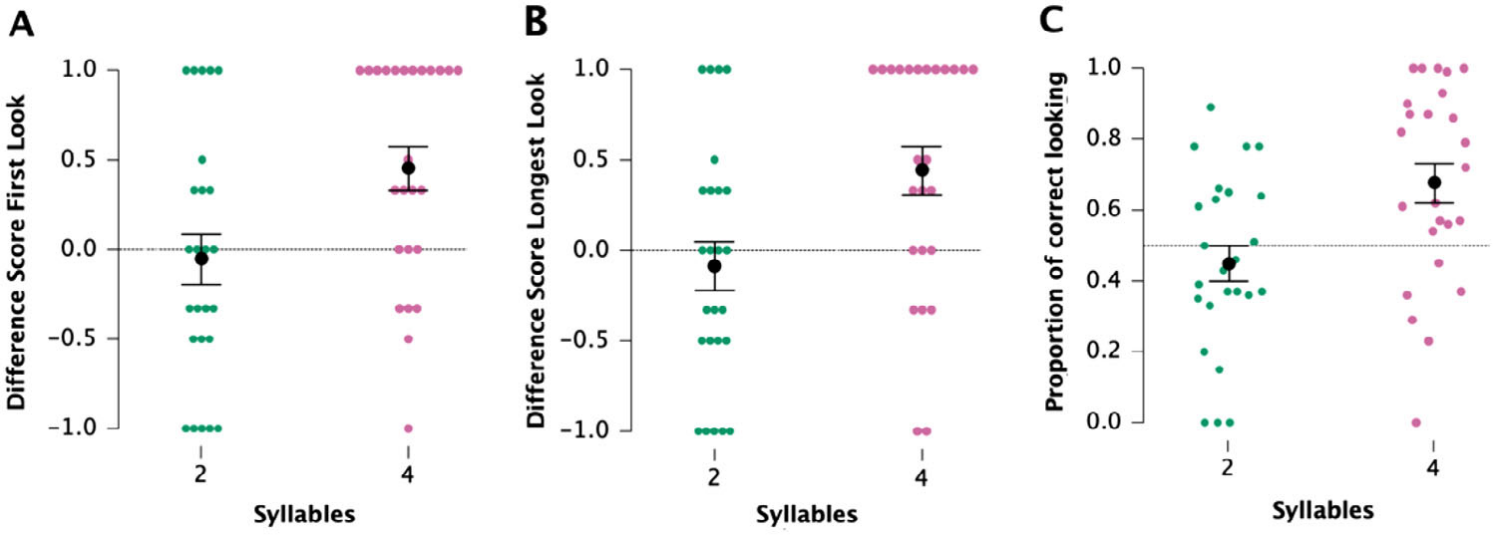
Main results of Condition 2—infants’ ability to discriminate between sets of 2 and 4 syllables. These findings are summarized as follows: (A) Normalized difference scores based on the first‐look measures. (B) Individual distribution of the normalized difference scores calculated on the longest look: Colored dots represent individual participants; black dots indicate the group means and bars the standard error. Chance is determined by the dotted black line in the middle. (C) Proportion of looking time (correct side).

The results of Condition 2 are depicted in Figure 3. The analysis considering the first look showed that, in the 2‐syllables sequence, infants’ mean difference score was −0.05, which did not differ from chance [*t*(24) = −0.38; *p* = 0.07; Cohen's *d* =–0.08]. For the 4‐syllable sequences, the infants’ mean difference score was 0.45, significantly greater than 0 [*t*(24) = 3.67; *p* = 0.001; Cohen's *d* = 0.74]. Moreover, a two‐tailed paired *t*‐test showed significant differences between the 2‐ and the 4‐syllable sequences [*t*(24) = −2.54; *p* = 0.02; Cohen's *d* = −0.51]. On average, infants looked at the center of the screen 74.5% of the time (in relation to the total number of trials). Their first look lasted 725 ± 222 ms on average. The mean duration of the longest look was 790 ± 206 ms. Registered mean response latencies were 776 ± 292 and 864 ± 271 ms for the 2‐ and 4‐syllable sequences, respectively.

Finally, the results regarding the longest look showed that infants’ mean difference score in the 4‐syllable sequences was significantly greater than chance [mean = 0.44; *t*(24) = 3.36; *p* = 0.003; Cohen's *d* = 0.67]. Infants did not perform above chance in the 2‐syllable sequences [mean = −0.09; *t*(24) = −0.65; *p* > 0.05; Cohen's *d* = −0.13]. A two‐tailed paired *t*‐test showed significant differences between the 2‐ and the 4‐syllable sequences [*t*(24) = −2.56; *p* = 0.017; Cohen's *d* = −0.51].

The data referring to the proportion of correct looking show that infants performed significantly above chance for the 4‐syllable sequences [mean = 0.68; *t*(24) = 3.15; *p* = 0.004; Cohen's *d* = 0.63] but were at chance for the 2‐syllable sequences, [mean = 0.45; *t*(24) = −1.03; *p* = 0.31; Cohen's *d* = −0.21]. A two‐tailed paired *t*‐test showed significant differences between the 2‐ and the 4‐syllable sequences [*t*(24) = −2.62; *p* = 0.015; Cohen's *d* = −0.52] in favor of the 4‐syllable sequences.

### 3.3 Comparison Between Conditions 1 and 2

**FIGURE 4:**
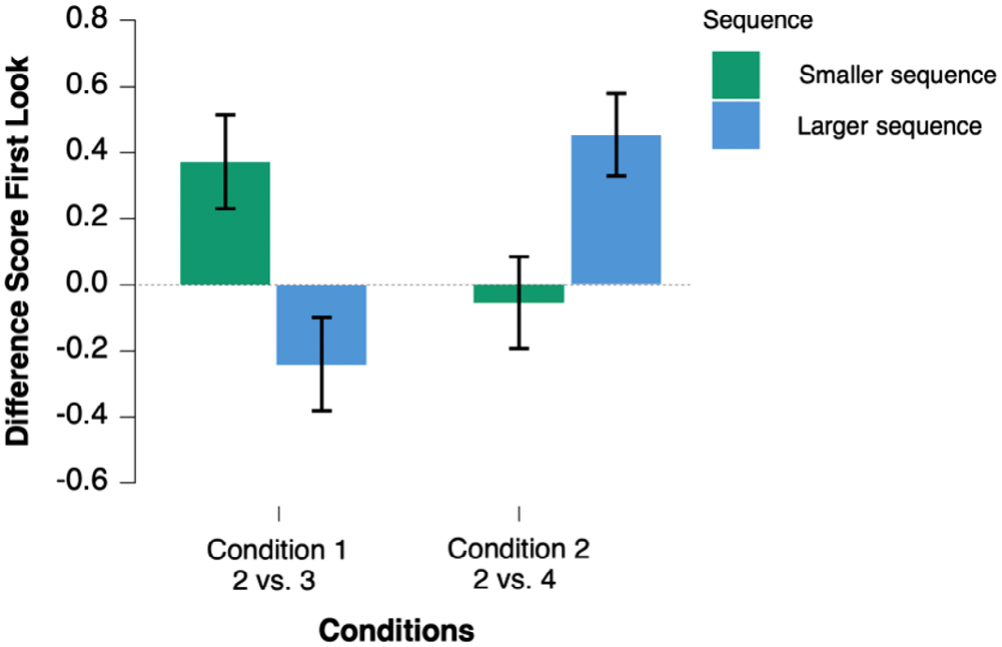
Comparison of the infants’ performance in Conditions 1 and 2. The *y*‐axis depicts mean difference scores. Error bars show standard errors of the mean.

A combined repeated‐measures ANOVA was carried out considering the first‐look data with experimental condition (Condition 1 vs. Condition 2) and set of syllables [smaller vs. larger sequence] as main crossed factors. The results indicated no main effect of sequence [*F*(1,24 = 0.18, *p* > 0.05, *η_p_* ^2^ = 0.008], or experimental condition [*F*(1,24) = 1.52, *p* > 0.05, *η_p_* ^2^ = 0.059], but a significant interaction between factors [*F*(1,24) = 8.43, *p* = 0.008, *η_p_* ^2^ = 0.260] as participants in Condition 1 generally performed better in the smaller than the larger sequences (mean estimates = 0.37 and −0.24 respectively), whereas in Condition 2, they generally performed better in the larger sequence than in the smaller sequence (mean estimate = 0.45 and −0.05, respectively). The results are depicted in Figure 4.

Moreover, in Condition 1, 17 out of the 25 participants performed above chance in the 2‐syllable sequence. Similarly, 17 infants performed above chance in the 4‐syllable sequence in Condition 2. Fourteen out of those 17 performed above chance in both conditions. A binomial test resulted in a *p* = 0.0064, indicating that the number of infants who were successful in both conditions is significantly different from a random proportion. This provides some hints for the stability of individual differences across conditions, as most of the successful infants performed well in both studies.

## 4 Discussion

The prevailing explanation of the boundary effect is that OTS, namely the numerical system used to process numbers up to 3, is incompatible with the ANS employed for larger numbers exceeding 3 (Feigenson and Carey 2005; Feigenson, Carey, and Hauser 2002; Hyde 2011; Mou and Van Marle 2014; Posid and Cordes 2015; Wang and Kibbe 2024). Here, we investigated the properties of the representational system used to encode syllables within the small number range and asked whether a boundary effect can also be detected when infants are presented with syllable sequences below and above 3.

Condition 1 confirmed previous studies by showing that infants successfully discriminate between 2‐ and 3‐syllable sequences when acoustic properties such as syllable identity, voice, pitch, and intensity were equalized across syllable sequences. Moreover, potentially confounding continuous variables such as individual item duration, interstimulus duration, and total duration varied across trials, thus leaving the number of syllables as the only reliable difference infants could use to distinguish the two sets. The infants’ ability to discriminate 2‐ and 3‐syllable sequences is convergent with results reported in previous number discrimination studies using linguistic stimuli (Benavides‐Varela and Reoyo‐Serrano 2021; Bijeljac‐Babic et al. 1993). Therefore, combined with earlier work, this study opens the possibility that 10‐month‐old infants possess an auditory event file analogous to the object file system in the visual domain. This system would be functional for precisely encoding speech stimuli and listing recently perceived speech sounds as separate items in working memory (for a compatible account, see Hauser et al. 2002).

Condition 2 contributes additional information regarding the properties of the representational systems operating over linguistic units. The results indicate that infants effectively distinguished 2‐ and 4‐syllable sequences, suggesting they have no trouble “crossing the boundary” between small and large numbers of syllables. Thus, differently from what has been reported in the visual domain, no catastrophic failures are observed when infants compare sequences greater versus smaller than 3.

Notably, the comparative analysis between Conditions 1 and 2 shows a contrasting pattern of responses. While in Condition 1, the 2‐syllable sequence appears easier for infants to learn compared to the 3‐syllable sequence, in Condition 2, the 2‐syllable sequence turns out to be more difficult with respect to the 4‐syllable sequence. These contrastive responses are relevant for at least two reasons. First, they indicate that infants effectively compared the two simultaneously presented sequences of each condition. The alternative, namely that infants focus only on one sequence type while disregarding all the other sounds, seems unlikely. If that was the case, the infants’ behavior should have been the same in both conditions (e.g., learning the 2‐syllable sequence), independently of the numerical features of the other sounds (i.e., either 3‐ or 4‐syllable sequences). Second, they provide a first indication that infants represent the syllable sets differently in the two conditions. At this point of our research, it is premature to draw firm conclusions regarding the nature of these representations. One possibility is that infants flexibly process sequences smaller or larger than 3 in either precise or approximate formats. This conjecture is substantiated by considering the features of the task. As stated in the introduction, a hallmark of discrimination in the two‐alternative working memory procedure is that monolingual infants learn one of the two sets, the one they find easier to process (Hochmann et al. 2011; Kovacs and Mehler 2009). In Condition 1, as expected, participants’ performance was above chance in the two‐syllables sequences because 2 items impose a lower load on working memory than 3 items. In Condition 2, discrimination was also attained, but infants found the 4‐syllable sequence less challenging, possibly because they represented it as one large ensemble rather than encoding each of the units that compose it separately. Thus, while in Condition 1, participants might have achieved discrimination by indexing individual sound files, in Condition 2, they might have represented supra‐span sequences as one large unit with approximate representations of the number of syllables.

Alternatively, infants might engage the use of ANS for both small and large number of syllables. This implies that infants achieve discriminability of the two sequences upon comparing the ratio, not the absolute difference between two values. This alternative is congruent with previous studies in the visual domain showing that, by 5 months of age, infants discriminate numerosities in a 1:2 ratio (as in Condition 2 of the current study) and by 9–10 months of age they are able to distinguish a 2:3 ratio, as in Condition 1 (e.g., Brannon, Suanda and Libertus 2007; Libertus and Brannon 2010; Lipton and Spelke 2003, 2004; McCrink and Wynn 2007; Wood and Spelke 2005; Wynn et al. 2002; Xu and Arriaga 2007; Xu and Spelke 2000). By accepting this interpretation, however, one should expect infants in the current task to learn either the smaller or the larger sequence across conditions, parsimoniously reflecting the use of the same system of representation. Conversely, a shift from learning the smaller sequence in the first condition to learning the larger one in the second condition challenges this account. As explained above, it is more likely that in certain situations infants flexibly engage different representations to deal with the task. However, further experiments are necessary to better characterize the mechanisms supporting the processing and discrimination of multisyllabic sequences and the conditions under which they are employed. Independently of the format they grasp, the results demonstrate that 9–10‐month‐old infants show simultaneous discrimination and processing of both small and large numbers of linguistic stimuli, as they probably do in real life.

Although previous studies have unveiled circumstances under which infants also succeed in comparing sets crossing the small‐large boundary (for reviews, see Hyde 2011; Mou and Van Marle 2014; Posid and Cordes 2015), there are two crucial aspects of the present results that are unique to this literature and provide novel insights regarding the functioning of the mechanisms underlying multiple item representations in infancy. First, previous investigations in the visual domain directly manipulated task parameters to facilitate the distinction. For example, researchers prevented catastrophic forgetting in infants exogenously by increasing the saliency of the encoded objects, adding individually distinctive features to each object, or spatial cues to parse larger arrays into smaller units (Feigenson and Halberda 2008; Rosenberg and Feigenson 2013; Zosh et al. 2011; Zosh and Feigenson 2015). In the present study, however, the stimuli themselves could not modulate the grouping or individuation abilities of the infants since the features and presentation style of the stimuli were identical in both syllable sequences within each condition. Because the only manipulation concerned information load, it is likely that a possible shift in the format of representation could also occur endogenously as the number of linguistic units and working memory demands increases (for compatible accounts, see Hyde 2011; Mou and Van Marle 2014). Second, previous studies controlled the to‐be‐discriminated stimuli to induce the same representational format. The manipulation was thought to avoid incompatibilities between the two systems. For example, some studies facilitated the precise representation of large sets >3 within the OTS (e.g., Feigenson and Halberda 2008; Zosh and Feigenson 2015), or the discrimination of large and small sets both represented as magnitudes via ANS (e.g., Cordes and Brannon 2009; Starr et al. 2013). Conversely, the present results indicate that, in the linguistic context, infants might not initially require small and large sets in the same representational format in order to compare them. In fact, the results of Condition 2 suggest that infants possibly represent 4 syllables as one large magnitude and 2 syllables as 2 separate units, with no interferences or failures to distinguish between them.

We argue that this flexible mechanism possibly enables infants to simultaneously maintain detailed linguistic information up to their typical memory capacity and coarse information beyond that limit (Räsänen et al. 2018; Zosh and Feigenson 2015). An emblematic example of the infants’ ability to represent arrays that contain vastly more items than their working memory can individually store can be observed in their extraction and encoding of rhythmic and prosodic units. It is well‐established that preverbal infants use these large ensembles to learn about morphosyntactic features of their native language(s). They facilitate grouping, segmenting, and breaking into the speech code, eventually providing infants access to more accurate representations of individual lexical, syllabic, and even sub‐syllabic units (Abboub et al. 2016; Benavides‐Varela and Gervain 2017; Hochmann et al. 2011; Gervain et al. 2020; Langus et al. 2017; Martinez‐Alvarez et al. 2023; Morgan 1996; Werker 2018). This capacity is clearly observed in the first months of life, well before the working memory capacities fully mature.

In conclusion, these results reveal the infants’ ability to flexibly deal with small and large numbers of multisyllabic sequences with no boundaries or incompatibilities between them. Infants’ capacity to simultaneously keep track of units of different magnitudes contributes to understanding how they successfully process, store, and learn from streams of speech sounds that lie outside the bounds of their working memory capacities.

## 5 Limitations

The current study focuses on the boundary effect in the auditory domain but provides only preliminary information regarding the actual size of phonological working memory in young infants. The data show direct proof that infants achieved results above chance with 2‐syllable sequences in Condition 1 and 4‐syllable sequences in Condition 2, pointing to the conceivable limit of 3 individual items in working memory. However, to determine infants’ ability to encode 3 auditory elements precisely, their ability to associate 3‐syllable sequences with a specific screen side must be tested. Furthermore, this study was conducted with 10‐month‐old monolingual infants only, leaving open the fundamental question of how these abilities emerge and develop at other ages and in multilingual environments. Finally, while the capacity to discriminate 2 versus 4 tones has been previously reported in infants (van Marle and Wynn 2009), it would be insightful to evaluate this contrast with the two‐alternative‐looking paradigm employed here to contribute a direct comparison of infants’ working memory capacities with linguistic and nonlinguistic stimuli.

## Author Contributions

Conceptualization: Silvia Benavides‐Varela and Natalia Reoyo‐Serrano. Methodology: Silvia Benavides‐Varela and Natalia Reoyo‐Serrano. Software: Natalia Reoyo‐Serrano and Anastasia Dimakou. Formal analysis: Natalia Reoyo‐Serrano and Anastasia Dimakou. Data curation: Natalia Reoyo‐Serrano. Investigation: Natalia Reoyo‐Serrano. Writing – original draft: Silvia Benavides‐Varela and Natalia Reoyo‐Serrano. Visualization: Natalia Reoyo‐Serrano. Writing – review and editing: Silvia Benavides‐Varela, Daniela Lucangeli, Tamara Bastianello, Chiara Nascimben, and Anastasia Dimakou. Funding acquisition: Silvia Benavides‐Varela. Resources: Silvia Benavides‐Varela, Daniela Lucangeli, and Natalia Reoyo‐Serrano. Supervision: Silvia Benavides‐Varela.

## Conflicts of Interest

The authors declare no conflicts of interest.

## Supporting information



Table S1. Sound sequences used in experimental trials.
Familiarization trials results.

## Data Availability

Data and materials can be found in our project on the Open Science Framework (OSF): https://osf.io/z7rwf/?view_only=d292e517f38341ce9a4c333727bc04bd.

## Endnotes

1 Young infants also succeed in discriminating exclusively small sets when redundant information is provided in tasks that include multimodal input. For example, infants differently look at displays where the number of visual items or faces is congruent or incongruent with the number of sounds or voices (e.g., 2 visual items and 3 sounds or vice versa; Starkey et al. 1983; Starkey et al. 1990; Kobayashi et al. 2005; Jordan and Brannon 2006). It is thus likely that the precision of the representations might differ when auditory stimuli is solely presented compared to when multiple types of information (visual, auditory, tactile) are available. Multimodal representations of individual entities (e.g., faces) could, arguably, increase the likelihood that the information will be more precisely encoded and tracked in working memory.
