# Supplementary material for: Crossing the Boundary: No Catastrophic Limits on Infants’ Capacity to Represent Linguistic Sequences
Source: Dev Sci. 2025 Apr 7;28(3):e70015. doi: 10.1111/desc.70015 (PMC11976043; doi:10.1111/desc.70015)
Supplement: Supplementary file 1 — Table S1. Sound sequences used in experimental trials. Familiarization trials results. [file DESC-28-e70015-s001.docx]

**Supplementary Materials**

**Familiarization phase**

This section presents the analyses performed over the familiarization of each Condition of the experiment.

**Condition 1**

*General pattern*

The overall pattern of responses considering all familiarization trials was similar to the one observed in the test phase. Infants’ mean difference score was above chance for the 2-syllable sequences [Mean = 0.31; t(23) = 2.85; p = .009; Cohen’s d = 0.58] but not for the 3-syllable sequences [Mean = -0.11; t(24) =-0.94; p = .35; Cohen’s d = -0.19].

*Pattern over time*

The analysis of the individual blocks shows that infants start performing significantly above chance in the 2-syllable sequence in blocks 2 and 3 (see below). In contrast, for the 3-syllable sequence, the absence of the effect is constant across blocks. The first-look data for the 2-syllable sequence are presented in Figures S1 and S2, and for the 3-syllables, Figure S2.

Infants anticipated the puppet's appearance on either side of the screen in 59.3% of the familiarization trials in the first block, 59.3% in the second block, 48.7% in the third block, and 31.3% in the fourth block.

*2-syllable sequences*

Infants’ mean difference score was above chance for the second [2^nd^ Block: Mean = .38; t(22) = 2.44; p = .023; Cohen’s d = .51] and the third learning blocks [3^rd^ Block: Mean = .38; t(21) = 2.18; p = .041; Cohen’s d = .46] but not for the first [1^st^ Block: Mean = .19; t(22) = 1.14; p = .27; Cohen’s d = .24] and last one [4^th^ Block: Mean = .13; t(15) = .49; p = .63; Cohen’s d = .12]. The latter is possibly due to attention depletion, as evidenced by the reduced number of participants providing data in this block.


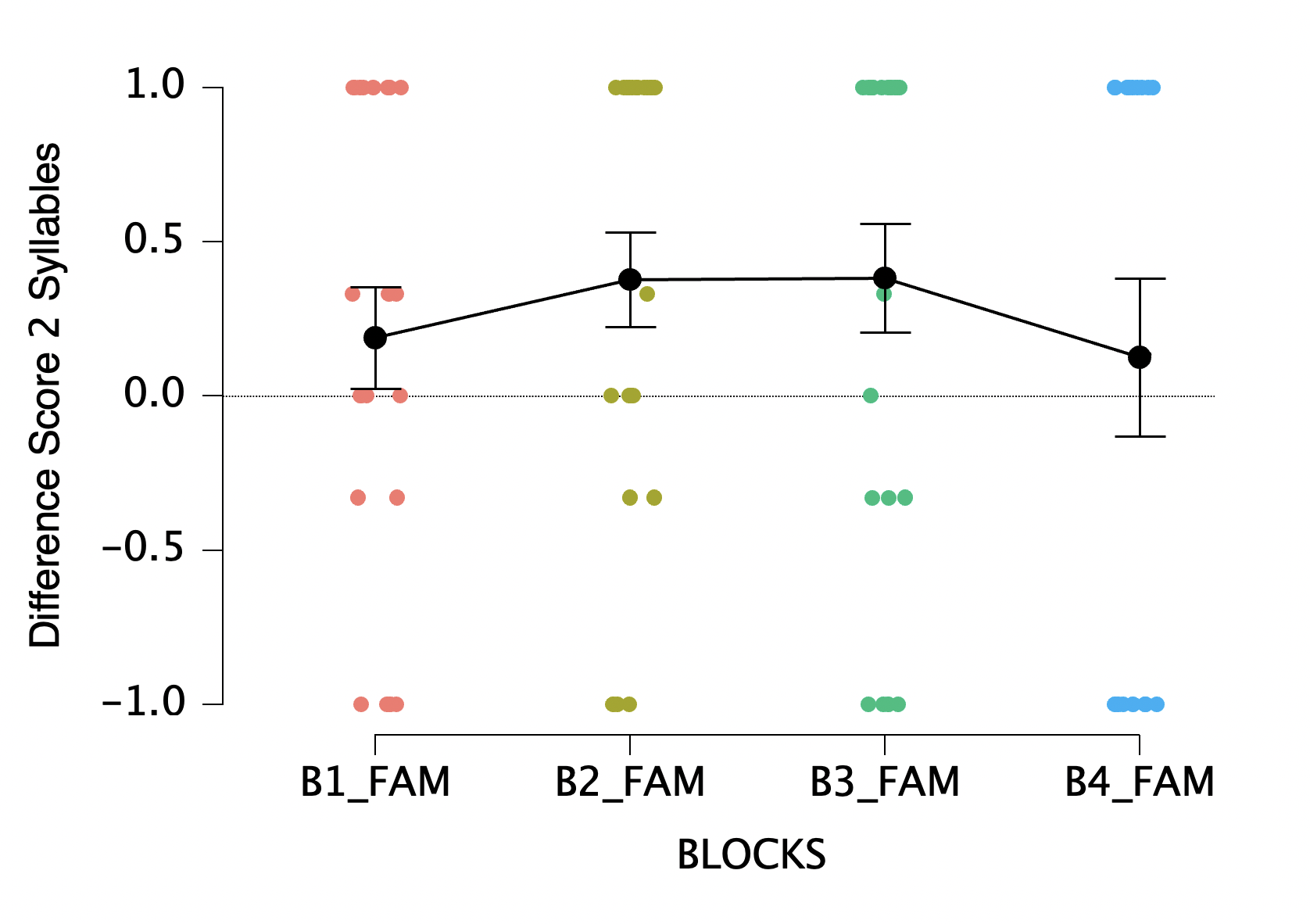


**Figure S1.** Normalized difference scores computed over the first look in the Familiarization phase of Condition 1 and for 2-syllable sequences. The y-axis shows the mean difference scores. Colored dots represent individual participants’ scores in each block, the black dot indicates the group mean, and the bars depict standard errors of the mean.

*3-syllable sequences*

Infants’ mean difference score was not above chance in any of the familiarization blocks [1^st^ Block: Mean = -.07; t(22) = -.38; p = .71; Cohen’s d = -.08; 2^nd^ Block: Mean = .05; t(21) = .29; p = .78; Cohen’s d = .06; 3^rd^ Block: Mean = .11; t(18) =.49; p = .63; Cohen’s d = .11; 4^th^ Block: Mean = -.13; t(15) = -.57; p = .58; Cohen’s d = -.14].

**
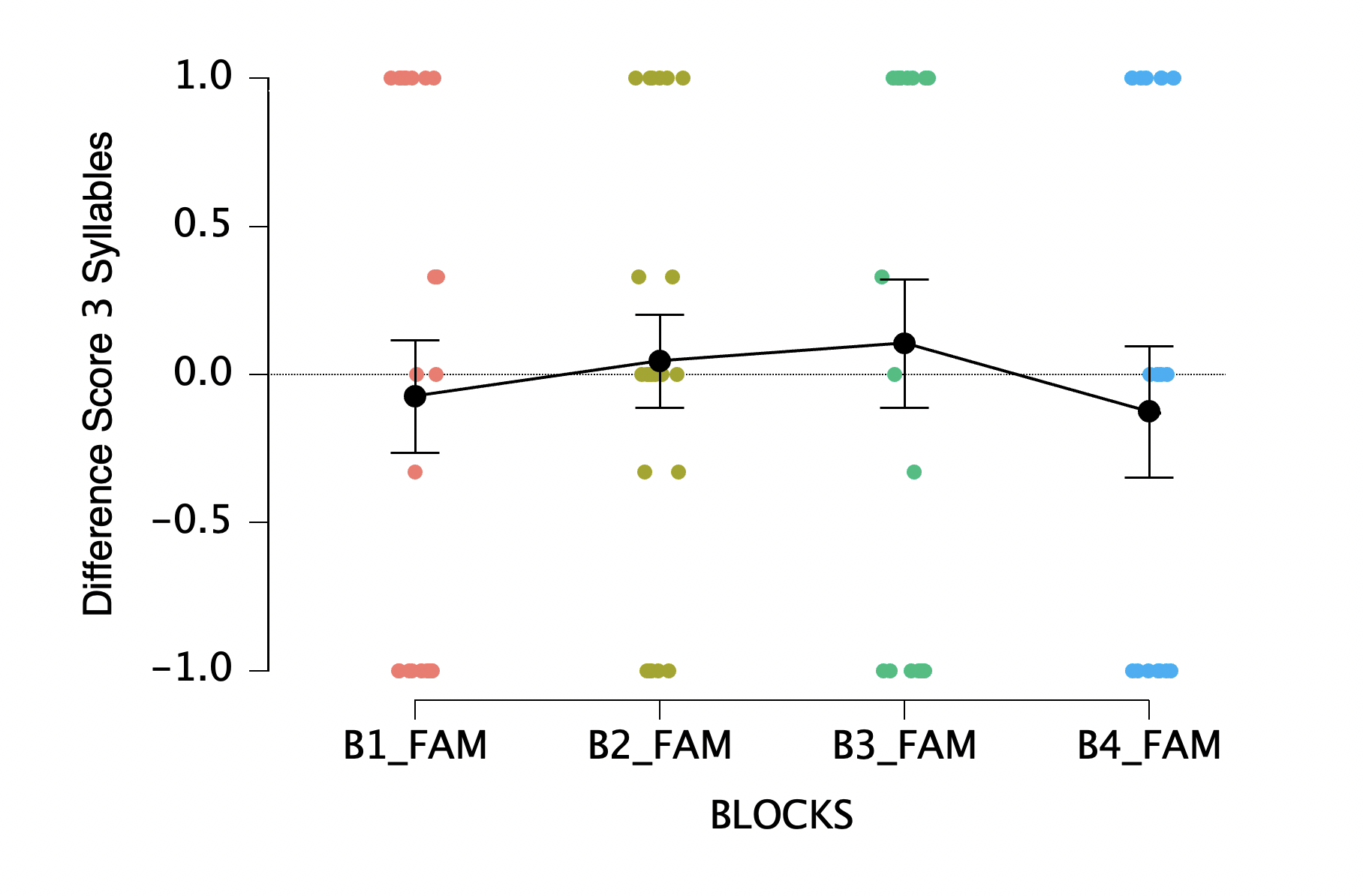
**

**Figure S2.** Normalized difference scores computed over the first look in the Familiarization phase of Condition 1 and for 3-syllables sequence. The y-axis shows the mean difference scores. Colored dots represent individual participants’ scores in each block; the black dot indicates the group means, and the bars depict standard errors of the mean.

**Condition 2**

*General pattern*

As in the test trials, infants’ mean difference score was not above chance in the 2-syllable sequences [Mean = -0.16; t(24) =-1.61; p = .12; Cohen’s d = -0.32]. On average, positive difference scores were registered in the 4-syllable sequences, but the overall difference against chance was not significant [Mean = 0.16; t(24) = 1.35; p = .19; Cohen’s d = 0.27].

*Pattern overtime*

Across time, infants initially had negative scores for the 4-syllable sequences, but the effect was progressively inversed, reaching significance (performance above chance) in the last blocks (see detailed analysis below). The first look data for the 2-syllable sequences are presented in Figures S3 and for the 4-syllables sequences in Figure S4.

Infants anticipated the puppet's appearance on either side of the screen in 58% of the familiarization trials in the first block, 48.7% in the second block, 49.3% in the third block, and 37.3% in the fourth block.

*2-syllable sequences*

Infants’ mean difference score was not different from chance in any block [1st Block: Mean = .32; t(18) = 1.14; p = .061; Cohen’s d = .46; 2nd Block: Mean = -.03; t(21) = 2.44; p = .89; Cohen’s d = -.03; 3rd Block: Mean = -.26; t(16) = 2.18; p = .23; Cohen’s d = -.30] except for block 4 [Mean = -.50; t(13) = .49; p = .023; Cohen’s d = -.69].

**
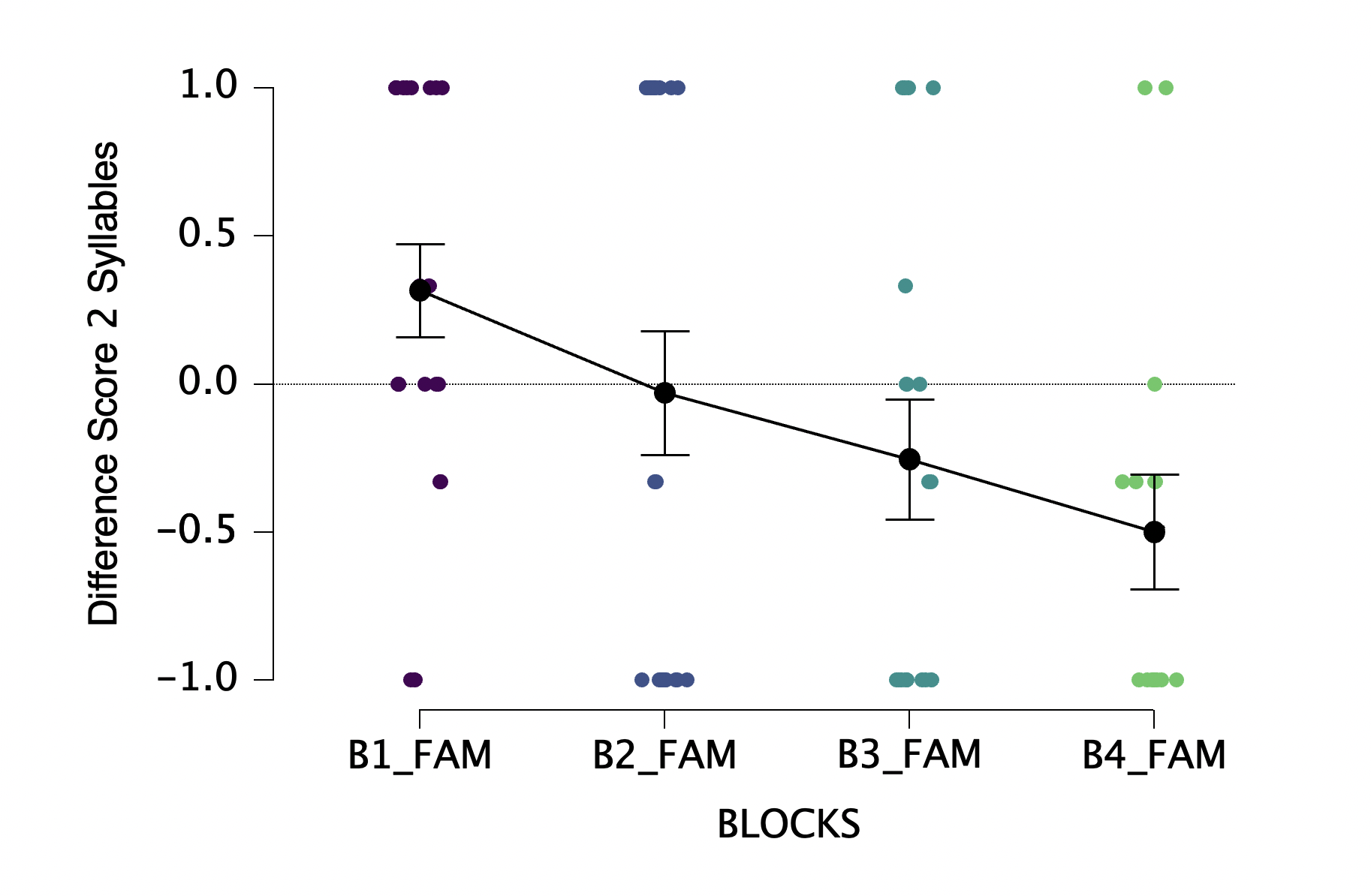
**

**Figure S3.** Normalized difference scores computed over the first look in the Familiarization phase of Condition 2 and for 2-syllables sequence. The y-axis shows the mean difference scores. Colored dots represent individual participants’ scores in each block; the black dot indicates the group means, and the bars depict standard errors of the mean.

*4-syllable sequences*

Considering the 4-syllables sequence, infants’ mean difference score reached performance above chance at the end of the experiment, namely in blocks 3 [ 3^rd^ Block: Mean = .45; t(22) = 2.18; p = .012; Cohen’s d = .57] and 4 [4^th^ Block: Mean = .47; t(18) = .49; p = .009; Cohen’s d = .67]. At the beginning of the study, in blocks 1 or 2, the performance was not above chance [1^st^ Block: Mean = -.20; t(21) = 1.14; p = .35; Cohen’s d = -.21; 2^nd^ Block: Mean = .33; t(16) = 2.44; p = .095; Cohen’s d = .43].

**
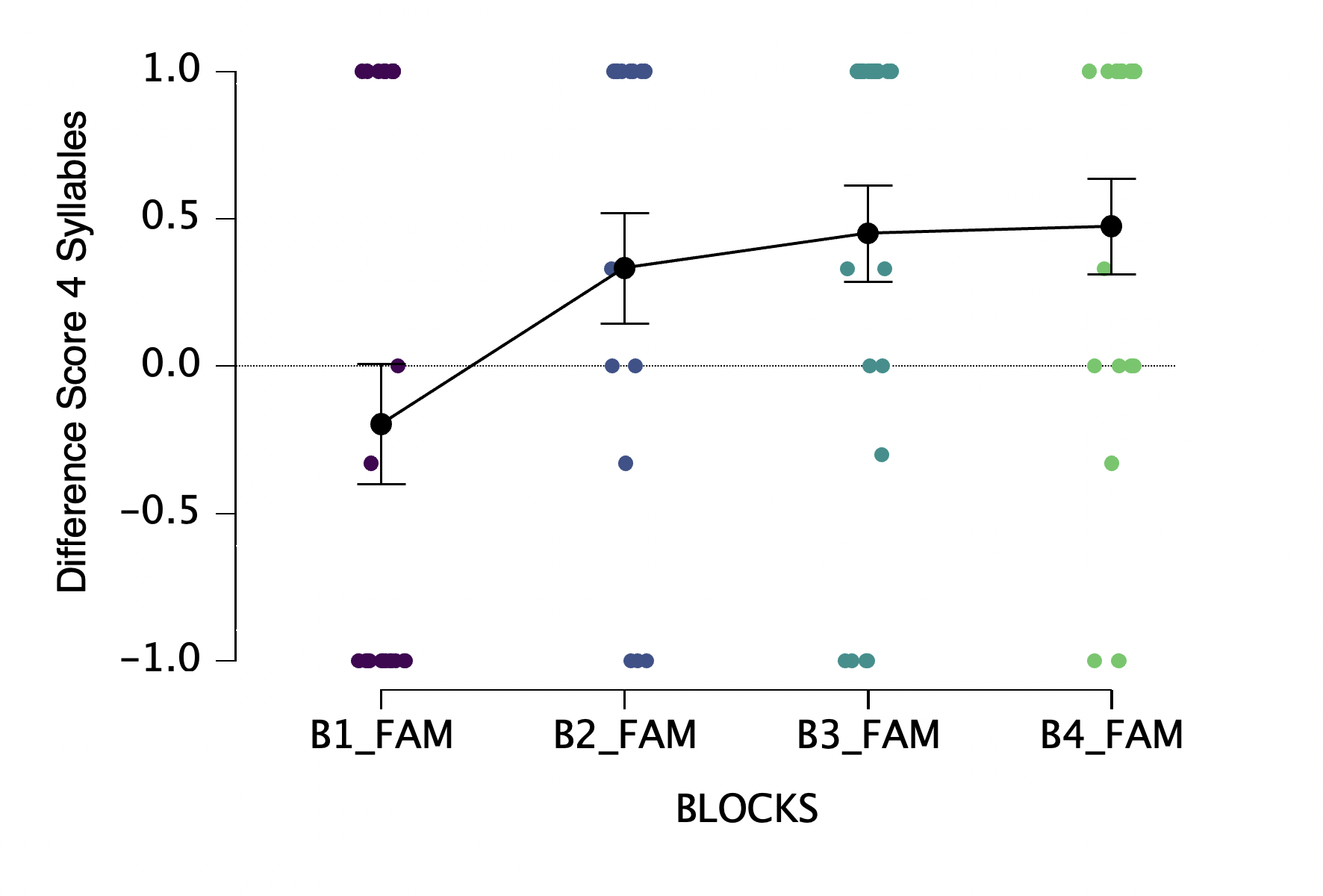
**

**Figure S4.** Normalized difference scores computed over the first look in the Familiarization phase of Condition 2 and for 4-syllables sequence. The y-axis shows the mean difference scores. Colored dots represent individual participants’ scores in each block; the black dot indicates the group means, and the bars depict standard errors of the mean.

*General considerations regarding Familiarization trials*

It is relevant to note that a key decision in the two-alternative-looking paradigm is how many familiarization trials to include and how to structure the test trials. In the present case, the training trials were interleaved with test trials in the form of rapid-learning blocks whereby -in line with adult definitions (Cowan et al., 2020)- the use of working memory is emphasized. Hence, this task approaches the highly complex nature of online processing and may provide additional hints regarding the extent to which infants organize multiple syllables in everyday contexts.

We warn the reader that data from familiarization trials might not be directly predicting infants’ performance in the test for at least two reasons: 1) the temporal interval during which infants could show an anticipatory look was only 1 sec, compared to the 2-sec window in the test trials and, as a consequence 2) infants anticipate on average in less than 50% of the trials, and their anticipations are not equally distributed across trials. Thus, while the dependent variables in some trials can be computed over a fair number of participants, other trials could include as few as 13 infants. Thus, the resulting curve might not be fully informative about the time course of learning because it shows the behaviors of different infants in different trials.

*Evaluation of side biases*

In some cases, it seems that, when participants are exposed to the unlearned quantity (3-syllables in Condition 1 or 2-syllables in Condition 2), they tend to look to the side corresponding to the learned quantity (2-syllables in Condition 1, or 4-syllables in Condition 2). This raises the question of whether side biases might be affecting the outcomes.

This possibility was explored using the following logic: If there were some infants that displayed side biases, then the proportion of first looks to one side (say left) should be significantly above or below chance (50%). We tested this by calculating the number of first looks each infant made to the left side overall and dividing these by the total number of valid trials they made. This allowed us to obtain the proportion of trials in which infants first looked to the left side. We then compared the distribution of these values against chance using a two-tailed T-test. The results indicate that infants looked first to the left side in 52% of the trials on average and that the distribution of the group did not differ from chance (p=.40). A similar logic was used considering the total looking time. In this case, the number of trials in which infants’ total looking time was longer to the left side was considered. The results showed that infants looked longer to the left side in 48% of the trials and that the distribution of the group did not differ from chance (p=.64).

We also explored potential side biases at the individual level by identifying outliers, namely infants whose distribution was 2 standard deviations below or above the average of the group. Only one participant showed a right-side bias (ID 1 in the Database). We then re-ran all the analyses excluding this participant and found that the results remained stable (both significant and non-significant differences were confirmed).

**Reference**

Cowan, N., Belletier, C., Doherty, J. M., Jaroslawska, A. J., Rhodes, S., Forsberg, A., ... & Logie, R. H. (2020). How do scientific views change? Notes from an extended adversarial collaboration. Perspectives on Psychological Science, 15(4), 1011-1025.
